# Parosteal Lipoma of the Thigh: A Report of a Rare Case and Review of the Literature

**DOI:** 10.7759/cureus.99681

**Published:** 2025-12-20

**Authors:** Kavita Y Shah, Suresh Phatak, Kajal Mitra, Prashant Onkar, Pranit B Pantawane

**Affiliations:** 1 Department of Radiodiagnosis and Imaging, NKP Salve Institute of Medical Sciences and Research Centre, Nagpur, IND

**Keywords:** ct scan, lipoma, lipomatous tumor, mri, periosteum

## Abstract

Parosteal lipomas are rare benign mesenchymal tumors consisting of mature adipocytes arising in close association with the periosteum. It accounts for less than 0.3% of all lipomas and is most often seen in the long bones of the extremities. These lesions are slow-growing and often asymptomatic, which can delay clinical recognition. Due to their proximity to bone, they can occasionally cause cortical remodeling or reactive bone changes, making radiological evaluation essential for an accurate and definitive diagnosis.

We report a case of a 51-year-old female who presented with a gradually progressive swelling over the medial aspect of the proximal thigh for one year. The patient denied any prior history of trauma or any surgical procedure. On local examination, the swelling was soft in consistency, non-tender, and immobile. The overlying skin was normal, with no ulceration, erythema, or discoloration, and there was no associated warmth or fluctuation. On functional assessment, there was no restriction in joint movements, and neurovascular examination of the limb was normal. Routine laboratory investigations were within normal limits.

Parosteal lipomas are rare entities that can be clinically mistaken for other soft-tissue or periosteal masses. Their characteristic attachment to the bone surface differentiates them from subcutaneous lipomas. Imaging modalities such as MRI play a crucial role in delineating their composition and relationship to adjacent structures. Surgical excision remains the treatment of choice, with an excellent prognosis and negligible recurrence risk. This report highlights a rare presentation of parosteal lipoma of the thigh in a 51-year-old female. Imaging findings can aid in timely diagnosis, prevent misclassification as a malignant lesion, and guide appropriate management.

## Introduction

Parosteal lipoma is an uncommon variant among lipomatous tumors. This lesion usually affects people between the ages of 40 and 60 years. The thigh is the most frequent site of involvement, followed by the forearm, calf, arm, and, less commonly, the ribs [1]. Although lipomas can occur anywhere in the body, they most commonly arise in the trunk or upper extremities. While parosteal lipomas are small and soft, some can be relatively hard and can grow to a considerable size. Giant lipomas are defined as lipomas measuring more than 10 cm in at least one dimension or weighing approximately 1 kg [2].

Lipomas in the femur tend to be larger than those occurring at other sites. Surgical excision is the treatment of choice for these lesions. Lipomas can develop in the parosteal, intraosseous, intramuscular, intermuscular, and subcutaneous areas. Before classifying a parosteal lipoma as entirely benign, malignant tumors must also be considered in the differential diagnosis. The primary objective of this study is to highlight the importance of an accurate diagnosis so that timely treatment can be initiated. Because clinical diagnosis of parosteal lipoma is challenging, radiologic investigations such as X-ray, CT, and MRI are essential for establishing a definitive diagnosis [3]. This case report describes a rare instance of parosteal lipoma of the thigh and demonstrates how imaging findings alone can provide a reliable diagnosis of the condition.

## Case presentation

**Figure 1:**
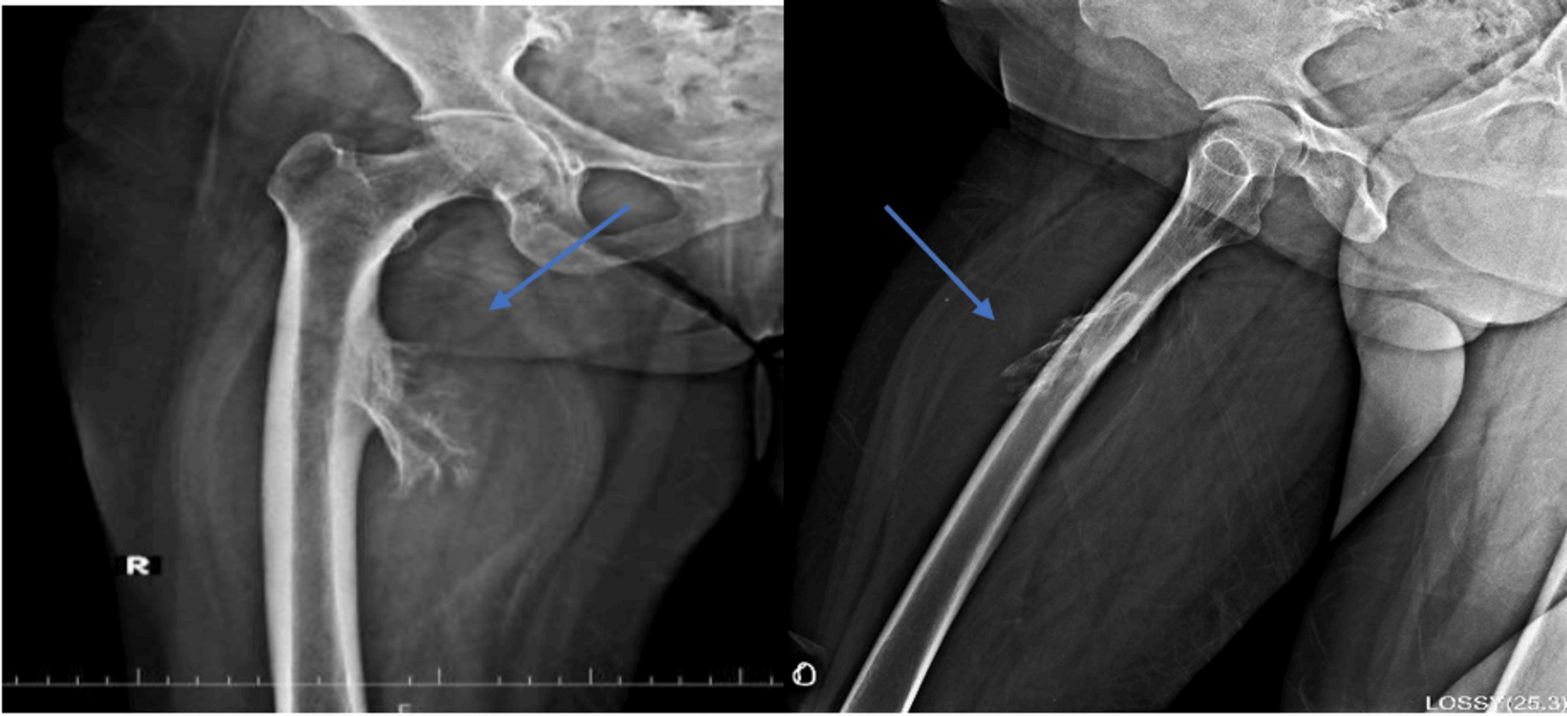
X-ray findings The images show a well-defined mixed-density mass with a few radio-opaque areas on the medial aspect of the proximal thigh, in proximity to the diaphysis of the right femur, with mild periosteal reaction. The cortex remains intact, with no medullary abnormality

**Figure 2:**
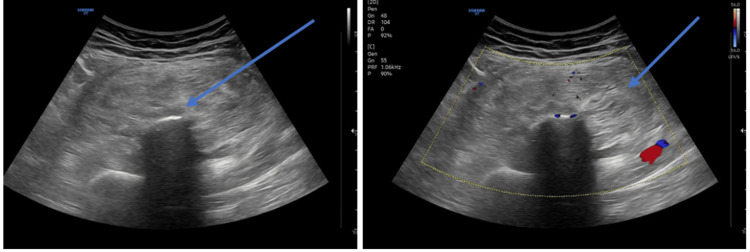
Ultrasonography findings The images show a well-defined isoechoic lesion with echogenic strands within and a few central calcifications. There is no evidence of vascularity on color Doppler

A 51-year-old female presented to OPD with complaints of swelling on the right thigh, present for one year, and gradually increasing in size. She reported no history of trauma or prior surgical intervention. On general examination, the swelling was soft, non-tender, and immobile. The overlying skin appeared normal, with no ulceration, redness, or discoloration. Functional assessment revealed no limitation of joint movements. For further evaluation, she underwent radiological investigations including X-ray, ultrasonography, non-contrast CT, and MRI. X-ray revealed a well-defined mixed-density mass with a few radio-opaque areas on the medial aspect of the proximal thigh, near the diaphysis of the right femur, along with a mild periosteal reaction. The cortex was intact, and no medullary abnormality was observed (Figure 1). Ultrasonography demonstrated a well-defined, isoechoic lesion with echogenic strands and areas of central calcification. Color Doppler showed no evidence of vascularity (Figure 2).

**Figure 3:**
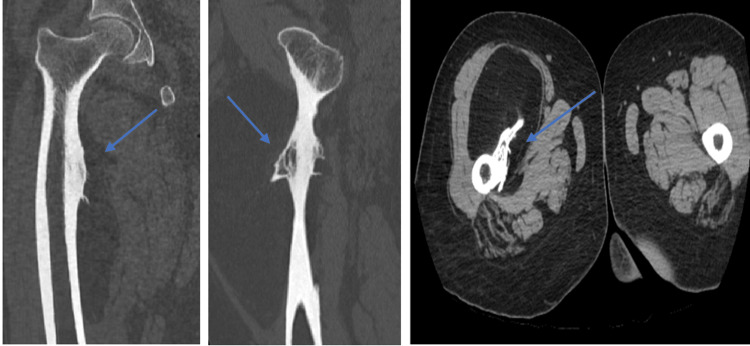
Plain CT scan (coronal, sagittal, and axial images) The images show a well-demarcated, intramuscular low-density (-110 to -70 HU) lesion on the medial aspect of the right femur with thick periosteal reaction and bone excrescence extending from the femur into the lesion. No communication with the medullary cavity is noted CT: computed tomography

**Figure 4:**
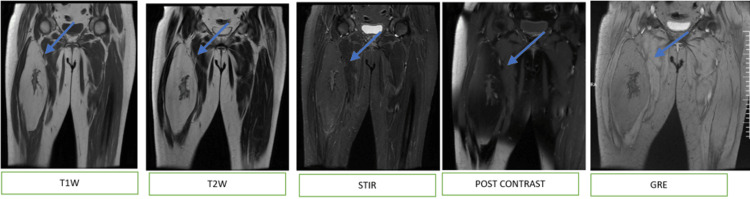
MRI findings The images show a well-circumscribed oval lesion noted abutting the cortex of the femur, appearing hyperintense to muscle on T1WI, T2WI, and suppressed on STIR. An ill-defined hypointense area noted within the lesion along the medial cortical margin of the proximal end of shaft of femur suggestive of osseous excrescences. Periosteal reaction noted along the cortical margin of the femur, with cortical thickening; however, no continuity with the medullary cavity is noted. On post-contrast, a few areas around the osseous excrescences show mild heterogeneous enhancement. The rest of the lesion shows no significant enhancement. There is no evidence of septations within the lesion. No associated solid component is observed. Additionally, there is no bone marrow edema or neurovascular invasion noted MRI: magnetic resonance imaging; STIR: short tau inversion recovery; GRE: gradient echo

On non-contrast CT, a well-demarcated, soft tissue, intramuscular low-density (-120 to -60 HU) lesion was observed on the medial aspect of the proximal thigh, near the diaphysis of the right femur, with a thick periosteal reaction and bone excrescence extending from the femur into the lesion. No communication with the medullary cavity was seen (Figure 3). Subsequently, MRI demonstrated a well-circumscribed oval lesion adjacent to the anteromedial cortex of the femoral diaphysis, displacing the surrounding thigh muscles. The lesion appeared hyperintense to muscle on T1WI and T2WI and was suppressed on STIR. Post-contrast images showed no evidence of enhancement (Figure 4).

## Discussion

Lipomas account for nearly half of all benign mesenchymal tumors, making them the most common soft tissue tumors. Histologically, lipomas are benign lesions composed of mature adipose tissue that closely resembles normal fat [4]. Parosteal lipoma was first described by Dr. Seerig in 1836 as a rare benign neoplasm originating from mature adipose tissue. In 1888, D’Arcy Power coined the term “parosteal lipoma” to highlight the tumor’s association with bone. The most common sites for parosteal lipomas include the femur, proximal radius, clavicle, humerus, tibia, and pelvis, although they can rarely occur in the ribs [5].

Parosteal lipomas exhibit unique imaging characteristics. On ultrasound, lipomas appear as hyperechoic lesions. Ultrasound is commonly used as an initial method to assess and evaluate a lipoma [6]. On X-ray, lipomas present as well-defined mixed-density masses. Surrounding the tumor, periosteal thickening and calcifications may also be observed. Additionally, osseous bending, projection, or smooth cortical scalloping can be seen [7]. On CT scans, parosteal lipomas appear as well-circumscribed, low-density masses (-110 to -70 HU) with no enhancement following contrast administration. MRI is particularly valuable for diagnosis, especially when malignancies such as liposarcoma are suspected [7]. Parosteal lipomas exhibit hyperintense signals on T1- and T2-weighted MRI images, similar to subcutaneous fat, and show low signal on fat-suppression sequences without contrast enhancement. MRI is especially helpful when nerve compression or involvement is present.

The differential diagnosis includes well-differentiated liposarcoma, parosteal (juxtacortical) osteosarcoma, periosteal chondrosarcoma, and osteochondroma. MRI with contrast can help differentiate among these entities. The most critical distinction is between well-differentiated liposarcomas and benign lipomas. Except for infiltrating lipomas, which might exhibit a different pattern, benign lipomas usually manifest as masses with regular edges. On MRI, the majority of benign lipomas show a uniform fat signal or fibrous components. Conversely, well-differentiated liposarcomas frequently exhibit lobulations or septations with a variety of non-adipose signals. On post-contrast MRI, these septations or lobulations show more enhancement than benign lipomas [8]. Features suggestive of a well-differentiated liposarcoma include size greater than 10 cm, fat content below 75%, and septa thicker than 2 mm. Parosteal (juxtacortical) osteosarcoma appears hypointense on T1-weighted and hyperintense on T2-weighted images, with variable post-contrast enhancement. It typically exhibits cloud-like bony growth with cortical invasion.

Periosteal chondrosarcoma is a lobulated, cartilage-rich tumor that exhibits high T2 signal intensity and a ring-and-arc enhancement pattern, often associated with cortical scalloping or bone loss. In osteochondroma, cortical continuity is maintained, with mild to moderate enlargement of the cartilage cap but no enhancement of the bony stalk [8]. The absence of medullary continuity, presence of fat signal intensity on MRI, and lack of contrast enhancement help differentiate parosteal lipoma from osteochondroma and liposarcoma. In this reported case of a parosteal lipoma in a 51-year-old female, the cortex remained intact, effectively ruling out periosteal chondrosarcoma, which usually induces cortical erosion [8]. Parosteal lipomas are associated with rearrangements of chromosome 12q13-15, particularly affecting HMGA2 (high-mobility group AT-hook 2). They also demonstrate a t(3;12)(q27-28;q13-15) translocation involving HMGA2 and LPP genes [9]. Treatment options for parosteal lipomas include either surgical excision or conservative management.

## Conclusions

We described a rare case of parosteal lipoma of the thigh in a 51-year-old female, with the diagnosis established entirely based on characteristic imaging findings. Radiography, CT, and MRI revealed a homogeneous fat-density juxtacortical lesion with periosteal reaction, cortical thickening, and osseous excrescences, but without soft-tissue nodularity, enhancement, or medullary involvement. These features are diagnostic of parosteal lipoma and allowed us to avoid unnecessary biopsy or invasive intervention. Early identification and imaging evaluation play a crucial role in distinguishing it from malignant lesions. This report highlights the importance of recognizing the typical imaging characteristics of parosteal lipomas, enabling accurate diagnosis and appropriate management. Complete surgical excision provides an excellent prognosis with minimal risk of recurrence.
